# Prevalence and Risk Factors of *Ascaris lumbricoides*, *Trichuris trichiura* and *Cryptosporidium* Infections in Elementary School Children in Southwestern China: A School-Based Cross-Sectional Study

**DOI:** 10.3390/ijerph15091809

**Published:** 2018-08-22

**Authors:** Dongjian Yang, Ya Yang, Yingjian Wang, Yu Yang, Shurong Dong, Yue Chen, Qingwu Jiang, Yibiao Zhou

**Affiliations:** 1 Department of Epidemiology, School of Public Health, Fudan University, 138 Yi Xue Yuan Road, Shanghai 200032, China; 17211020111@fudan.edu.cn (D.Y.); yayang16@fudan.edu.cn (Y.Y.); 17211020008@fudan.edu.cn (Y.W.); 16211020071@fudan.edu.cn (Y.Y.); 17211020051@fudan.edu.cn (S.D.); jiangqw@fudan.edu.cn (Q.J.); 2 Key Laboratory of Public Health Safety, Fudan University, Ministry of Education, Shanghai 200032, China; 3 Center for Tropical Disease Research, Fudan University, Shanghai 200032, China; 4 School of Epidemiology and Public Health, Faculty of Medicine, University of Ottawa, Ottawa, ON K1G 5Z3, Canada; Yue.Chen@uottawa.ca

**Keywords:** *A. lumbricoides*, *T. trichiura*, *Cryptosporidium*, school children, China

## Abstract

*Background*: Intestinal parasitic infections pose great public health challenges in school children in developing countries. The aim of this study was to assess the prevalence of *A. lumbricoides*, *T. trichiura* and *Cryptosporidium* among elementary school children in rural southwestern China. *Methods*: A school-based cross-sectional study involving 321 elementary school children was conducted in 2014 in the southwest of China. They were invited to provide a stool sample and interviewed about the sanitary situation and hygiene behavior. Stool specimens were examined for *A. lumbricoides* and *T. trichiura* using the Kato-Katz fecal thick-smear technique. The presence of *Cryptosporidium* was determined using a modified acid-fast staining method. *Results*: The prevalence of infection was 10.0% (95% CI: 6.9–13.8%) for *A. lumbricoides*, 25.2% (95% CI: 20.6–30.4%) for *T. trichiura* and 2.4% for (95% CI: 1.1–4.9%) *Cryptosporidium*. The prevalence of co-infection was 3.7% (95% CI: 1.9–6.4%) for *A. lumbricoides*/*T. trichiura*, 0.3% (95% CI: 0–1.7%) for *A. lumbricoides*/*Cryptosporidium* and 0.9% (95% CI: 0.2–2.7%) for *T. trichiura*/*Cryptosporidium*. Children from households using well or river water were associated with a greater odds of *A. lumbricoides* infection (aOR = 2.61, 95% CI: 1.12–6.05). Having a household lavatory was associated with a lower odds of *T. trichiura* infection (aOR = 0.50, 95% CI: 0.30–0.84). Children who had three meals at the school canteen on week days were at a lower risk of *Cryptosporidium* infection. The use of spring water as a water source was associated with lower odds of any intestinal infection (aOR = 0.56, 95% CI: 0.35–0.91). *Conclusions*: Our study calls for an intervention program of school-based deworming combined with health education, hygiene promotion and provision of safe water and improved sanitation.

## 1. Introduction

Infections with helminths (e.g., *Ascaris lumbricoides*, *Trichuris trichiura* and hookworm) and intestinal protozoa (e.g., *Cryptosporidium* and *Giardia intestinalis*) are global public health threats and are closely associated with poverty, unsafe water and inadequate sanitation and hygiene [1]. More than one billion people worldwide are infected with one or more species of soil-transmitted helminths (STH), of which over 267 million pre-school children and over 568 million school children are considered at risk of morbidity, especially in less developed countries [1,2,3]. STH infections fall within the grouping termed as neglected tropical diseases (NTDs), and nearly 70% of the global burden it causes occurs in Asia [4,5]. Infections can cause diarrhea, abdominal pain, malnutrition, physical and intellectual growth retardations [1]. Cryptosporidiosis is a major cause of diarrheal disease in developing and developed countries, and epidemiological studies have shown that *Cryptosporidium* is more prevalent in developing countries (5% or higher) than in developed countries (3% or less) [6]. *Cryptosporidium* was identified to be the second only to rotavirus as a cause of moderate-to-severe diarrhea in children during the first two years of life [7]. Infection with *Cryptosporidium* can lead to self-limiting diarrhea in immunocompetent individuals but life-threatening and prolonged diarrhea in immunocompromised ones such as people infected with HIV [8]. *Cryptosporidium* infection is also associated with malnutrition and growth deficits in children. The types of organisms, risk factors and routes of transmission of the three infectious diseases are different, but their transmission is related to poor sanitation conditions and sanitary practices (Table 1) [9].

Historically, infections of *A. lumbricoides* and *T. trichiura,* along with other intestinal helminth parasites, have been a major public health problem in China. There have been two national surveys of parasitic diseases conducted in China. The prevalence of infection was 47.0% for *A. lumbricoides* and 18.8% for *T. trichiura* in the first national survey of parasitic diseases (1988–1992), which dropped dramatically to 12.7% and 6.1% at the second national survey (2001–2004) [10]. Although, the prevalence of STH infections continuously decreased according to national surveillance data, *A. lumbricoides* and *T. trichiura* infections remain high in several underdeveloped regions in China [11,12,13,14]. A review of South and Southeast Asia demonstrates that school children have higher infection risks of *A. lumbricoides* (25%, 95% CI: 16–31%) and *T. trichiura* (22%, 95% CI: 14–34%) than the general population [15]. However, *Cryptosporidium* infection had been largely neglected and few studies had been conducted in the Yi ethnicity in SW China. Similarly, there are few studies on the epidemiology of STH infections and risk factors for infection in primary school children. Studies of *A. lumbricoides*, *T. trichiura* and *Cryptosporidium* in children are relevant to the control of these three infectious diseases because primary school children are an important reservoir of infection and at risk of morbidity. The aim of the present study was to assess the prevalence of infection for *A. lumbricoides*, *T. trichiura* and *Cryptosporidium*, and associated risk factors among elementary school children in this region, to provide a scientific basis for the formulation of government health policies and the promotion of school health education to reduce the prevalence of the three parasitic diseases in school-age children.

## 2. Materials and Methods

### 2.1. Study Area

**Figure 1:**
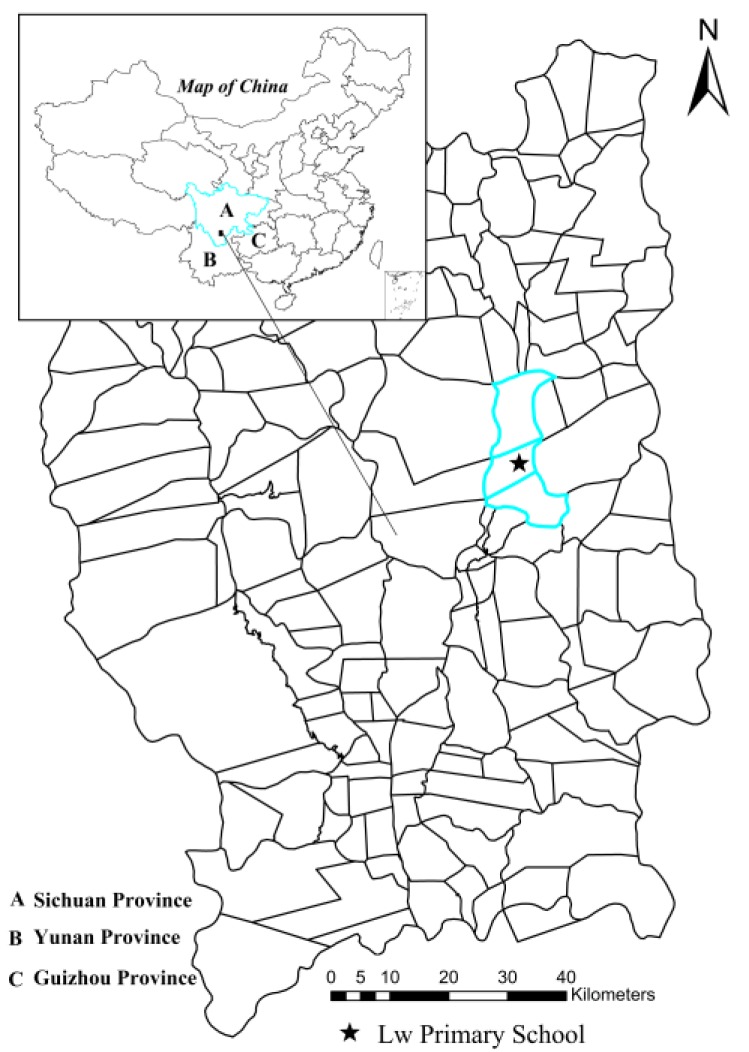
Map of the study area, showing Lw Primary School in Lw town, Puge County.

The survey was conducted from 23 October to 3 November 2014 in Lw Primary School (Figure 1), Lw town, Puge County of the Liangshan Prefecture, southwestern China. Lw town features a complex topography of mountains and valleys with an average elevation of 1800 m. The regional climate is subtropical monsoon, characterized by mild winters and warm and humid summers. The annual rainfall is 900 mm with the main rainy season from May to October and occasional rain throughout the rest of a year. The average temperature for the region is approximately 20 °C. The local population is approximately 3000, most of whom are of the Yi ethnicity. The region is one of China’s state-level poverty-stricken areas. Agriculture and animal husbandry are vital sources of household income. Unprotected contact with animals often causes local people to suffer from various zoonotic diseases.

At the time of the study, Liangshan Prefecture had poor sanitary conditions, especially at home, characterized by unsterilized drinking water and inadequate access to lavatories.

### 2.2. Participant Recruitment and Field Study Procedures

Schools are convenient sites for field surveys and school-age children are at high risk of helminths and protozoan infections. Lw Primary School of Lw town was selected with the help of the local health officials and all children from Grades 3, 4, 5 and 6 were selected for the survey. These four grades are selected because they are old enough have sufficient ability and good compliance to understand and complete the questionnaire. School teachers and the local health officials of the Center for Disease Control and Prevention (CDC) were invited to a sensitization meeting prior to our surveys. The study procedures and the structure of the questionnaire were explained in detail under the guidance of a unified protocol at the meeting. Health workers and teachers then informed all potential participants based on the protocol list and carefully explained the research objectives, procedures, and potential risks. A written informed consent form was obtained from the parents/guardians of the eligible students. A questionnaire and a clean plastic bag were distributed to the students by school teachers. Trained workers asked all participants carefully to ensure that each question was answered. Each participant was given a unique identification number.

### 2.3. Laboratory Procedures and Survey Measures

Stool samples were collected once for each participant and sent to the laboratory of the local CDC as soon as possible. For the diagnosis of helminth infection, three Kato-Katz thick smear slides were prepared within 24 h post-collection. Another two thin smears were prepared using modified acid-fast staining to detect the oocysts of *Cryptosporidium*. All the slides were read by two independent examiners and a third examiner was called in if there was a disagreement. The survey included information on demographic characteristics, risk factors, and sanitation conditions, which were associated with those three infectious diseases. Demographic characteristics data of school children included gender, ethnicity, age, and the number of family members. Risk factors of infections were based on four major items, including washing hands, eating raw food or drinking raw water, the place to eat, and engaging in farm work. Sanitation associated with the student’s home were assessed by two items, including water sources and types of lavatories.

### 2.4. Ethical Considerations

This study was approved by the Ethics Review Committee of the Ethical Institute of the School of Public Health, Fudan University (reference No. #2010-08-0235). After the survey, all children with infection were offered free anti-helminthic treatment according to the national guidelines.

## 3. Statistical Analysis

Data were checked and entered into EpiData version 3.1 (EpiData Assoc., Odense, Denmark) and internal consistency checks were done. Statistical analyses were carried out with the SAS version 9.4 software (SAS Institute, Cary, NC, USA). Descriptive summary of the children’s characteristics was computed as appropriate. Prevalence of *A. lumbricoides* and *T. trichiura* and *Cryptosporidium* infections with the 95% confidence interval were calculated. Fisher’s exact test was used to compare the prevalence of the three parasitic diseases in different age groups. Univariate and multivariate analyses were performed to identify risk factors for the three parasitic diseases and any intestinal infection (any intestinal infection refers to the infection with one or more of those three infectious diseases). In the univariate analyses, Pearson’s *χ*^2^ test was used to examine the associations of participants’ characteristics with *A. lumbricoides* and *T. trichiura* and *Cryptosporidium* infections by computing crude odds ratios (ORs) with 95% confidence intervals (CIs). A subsequent multivariate logistic regression model was employed to identify risk factors and adjusted ORs with 95% CIs were calculated. Since a correlation existed between living on campus and eating at the school canteen, have a household lavatory and types of lavatories at home, only one of the two related variables was included in the multivariate model at a time. A two-sided *p*-value of 0.05 or less was regarded as significant.

## 4. Results

There were a total of 348 eligible participants in the study. After exclusion of participants who did not provide feces or complete the questionnaires, 321 (92.2%) participants were included in the analysis. Demographic data showed similar distributions for those included and not included in the analysis (Table 2). 

Table 3 shows the prevalence of *A. lumbricoides*, *T. trichiura*, *Cryptosporidium* and coinfections by age group. The prevalence of infection was 10.0% (95% CI: 6.9–13.8%) for *A. lumbricoides*, 25.2% (95% CI: 20.6–30.4%) for *T. trichiura* and 2.4% (95% CI: 1.1–4.9%) for *Cryptosporidium*. The prevalence of co-infection was 3.7% (95% CI: 1.9–6.4%) for *A. lumbricoides*/*T. trichiura*, 0.3% (95% CI: 0–1.7%) for *A. lumbricoides*/*Cryptosporidium* and 0.9% (95% CI: 0.2–2.7%) for *T. trichiura*/*Cryptosporidium*. No triple infection was found in our study. The prevalence of *A. lumbricoides* infection differed among the age groups and was the lowest among children over 13 years of age (*p* = 0.04), while *T. trichiura* and *Cryptosporidium* infections and all the co-infections were not significantly different among the age groups (Table 3).

The characteristics of the participants are described in Table 4. The mean age of participants was 12.6 years (SD: 2.0). Among 321 children recruited, 62.93% were male; 98.75% were Yi people; 89.10% lived on campus and had their three meals of week days at the school canteen. At homes of the 321 participants recruited, 85.98% of the drinking water came from mountain springs, 60% of household lavatories were simple, and 30% were triple compartment or biogas pools.

Table 5 and Table 6 show the results of risk factors associated with *A. lumbricoides*, *T. trichiura* and *Cryptosporidium* infections. Children from households using well or river water were at a greater risk of *A. lumbricoides* infection (aOR = 2.67, 95% CI: 1.15–6.20). Having a household lavatory was negatively associated with *T. trichiura* infection (aOR = 0.50, 95% CI: 0.30–0.84). Children having their three meals at the school canteen were at a lower risk of *Cryptosporidium* infection (aOR = 0.06, 95% CI: 0.01–0.40). Similarly, living on campus was also a protective factor for *Cryptosporidium* infection (cOR = 0.11, 95% CI: 0.03–0.46). Using spring water as water source was a protective factor for any intestinal infection (aOR = 0.52, 95% CI: 0.28–0.99).

## 5. Discussion

This study reported the prevalence of *A. lumbricoides*, *T. trichiura* and *Cryptosporidium* infections in the elementary school children. The prevalence of *Cryptosporidium* infections in our study (2.40%) was comparable to the provincial estimate (2.96%) in Sichuan Province, China [15]. The prevalence of *A. lumbricoides* infection (10.0%), was similar to the national prevalence (12.7%) of the second national important parasitic disease survey from 2001 to 2004, but was lower than those of Guizhou Province (42.0%) and Sichuan Province (27.7%) [13]. Our study area is located in the southwestern of Sichuan Province, close to Yunnan and Guizhou Province (Figure 1). The reduced prevalence of *A. lumbricoides* infections may be related to the government’s deworming treatment of *A. lumbricoides* in high-risk populations. A survey of school-age children in 2010 also showed a reduction in the prevalence of *A. lumbricoides* infection in rural southwestern China [18]. For *T. trichiura* infection, the prevalence in our study (25.2%) was higher than both second national survey (4.6% in China Mainland) and the survey of school-age children in southwestern China in 2010 (14.3% in Guizhou Province, and 2.2% in Sichuan Province) [13,18]. According to the WHO report, the prevalence of STH infection in China was estimated to be less than 20% [22]. The prevalence of *T. trichiura* infections in the study area remained high. The main reason may be the unsatisfactory efficacy of currently recommended drugs against *T. trichiura* infection, especially when they are not regularly taken [23]. Poor hygiene, especially at home, may be another reason for the high prevalence of *T. trichiura* infection. Most children in the school drank unboiled water (84.42%) and ate raw food without washing (86.60%). Water, food, soil and hands are easily contaminated by worm eggs in the case of inadequate sanitation facilities and feces management, unsafe water and poor hygiene practices [24]. Therefore, improving access to adequate water, sanitation and hygiene (WASH) at the household level and in school is essential to prevent infections of *T. trichiura* [25]. In addition, the climatic factors in Liangshan Prefecture are within the plausible limits of STH transmission [5]. As expected, the prevalence of *A. lumbricoides* infection varied by age. However, the age patterns of *T. trichiura* and *Cryptosporidium* infections showed little variation, which probably reflected a combination of behavioral and socioeconomic factors [26]. Indeed, older students had more opportunities to receive health education and knowledge about infection prevention. Interestingly, although most hygiene habits tended to improve with age in our study, older students were prone to eating raw food. This finding emphasized the importance of continuing and strengthening health education programs targeting school children to reduce the intensity of parasites and the attendant morbidity [12].

A recent meta-analysis of the effects of water, sanitation, and hygiene (WASH) on STH infections reported that access to piped water and proper water treatment was associated with lower risks of *A. lumbricoides* and *T. trichiura* infections [27]. Our study found that using river or well water was associated with an increased risk of *A. lumbricoides* infection. In the study area, the main sources of drinking water in the study area were mountain springs (far from human living areas, piped after extensive precipitation and filtration), followed by wells or rivers (both close to living areas and susceptible to fecal pollution). Moreover, some students’ homes (14.33%) used river or well water as the source of drinking water and 84.42% of school children were in the habit of drinking unboiled water. The drinking water is rarely disinfected and is often contaminated by untreated infectious human excreta, which puts the local residents at great risk of intestinal parasitic infections. Safer drinking water is an urgent need in the rural regions of Liangshan Prefecture. Having a household lavatory was negatively associated with *T. trichiura* infection, which was similar to previous findings [19,27,28,29]. In order to reduce intestinal infectious diseases, the local government promoted and constructed triple compartment toilets or biogas digesters that can fully ferment feces to kill the eggs and ensure that human waste is safely separated from human contact in some rural areas [30,31]. But many families only have simple lavatories that pile up human stools and urine as a direct fertilizer. A cluster-randomized trial undertaken in Kenya demonstrated that school hygiene and sanitation reduced the re-infections of STH after school-based deworming [2]. A recent meta-analysis has shown that adequate sanitation is associated with the reduction of STH transmission risk (between 40% and 50% reduced infection rates) [28]. There is no doubt that improvements to water, sanitation, and hygiene (WASH) lead to a significant reduction in STH infection. Schools are available platforms, not only for health education but for sanitation improvements. School-age children are more likely to receive health education and tend to integrate new health behaviors into their daily lives [32].

Soil-transmitted helminthiasis, a group of NTDs, is thought to be associated with malnutrition and iron deficiency anemia, and has a negative impact on children’s physical and cognitive development [33]. Hence, reducing risk factors and regular deworming become important strategies for controlling STH [34]. Effective control of STH can have a positive outcome on students’ education, as chronic helminth infection impedes cognitive development and leads to growth delays, thus delaying enrollment and grade progression [35].

*Cryptosporidium* is typically transmitted by fecal-oral routes, through ingestion of human or domestic animal fecal-contaminated food or untreated water. In our study, students having meals at the school canteen or living on campus had lower odds of obtaining *Cryptosporidium* infection. Students who live on campus (89.10%) ate three meals at the school canteen. The elementary school we selected was basically a boarding school. The majority of the students stayed in the school dormitories for 5 days per week and returned home during holidays and weekends. One possible reason for the association of *Cryptosporidium* infection with eating in the school cafeteria was less chance of contact with domestic animals. Raising domestic animals is known as a risk factor for *Cryptosporidium* infection [36,37]. Another possible reason was that schools had better sanitation facilities and safer water, protecting those students who did not have access to these facilities at home [14,32,38]. The local government has been concerned about the safety of school drinking water. At Lw Primary School, improved water and sanitation facilities, including basic tap water, were available to the students. Tap water was used for handwashing without soap. A recent review showed that diarrhea and gastrointestinal diseases decreased as schools had access to adequate sanitation [39]. Improper waste disposal and unsafe water, especially at home, will increase the chance of transmission of fecal-oral diseases [14,40].

We recognize a number of limitations in our study. The diagnoses of three intestinal parasitic infections were based on a single stool sample and underestimation of the infection rates might be possible. There was also a lack of data on helminth infection intensities and *Cryptosporidium* genotypes. Further research is needed to determine the infection intensities of *A. lumbricoides* and *T. trichiura* and also the routes of transmission of *Cryptosporidium* among school children in this region. To truly understand the motivators for behavior and exposure to infection, further studies might be needed to systematically assess risk factors of intestinal parasites by using a combination of quantitative and qualitative methods including water quality monitoring and WASH spot checks [40]. The sample size is not enough for more accurate statistical analysis. In view of these limitations, the findings should be interpreted discreetly.

## 6. Conclusions

In summary, our study revealed a high burden of *A. lumbricoides* and *T. trichiura* infections among elementary school children in southwestern China. School-level interventions involving deworming, health education, improvement of environmental sanitation and hygiene and access to safe water might be required. Schools are a good platform for health education, not only for students but also for their families. New methods of health education (e.g., comic and animation) might be explored. There is no doubt that improving health awareness among students can reduce their risky behaviors related to parasitic infections. Similarly, we should actively promote the reconstruction of harmless lavatory (e.g., triple compartment or biogas) in rural areas and improve the rural sanitary conditions, to reduce the transmission of intestinal infectious diseases.

## Figures and Tables

**Table 1:** Compilation of *Ascariasis*, *Trichuriasis* and *Cryptosporidiosis* with relevance in Asia and China.

Disease	Organism Types	Disease Agent	Transmission Pathway Related to Water or Soil	Risk Factors	Sanitation and Hygiene	Relevance
*Ascariasis*	*Secernentea*	*Ascaris lumbricoides*	Fertilized eggs in the moist soil become infectious about two weeks later. Humans are infected through contaminated water and soil [16].	Preschool children and school age children; Hypoimmunity; Overcrowding; Poverty; Using stools to make fertilizer; Poor health education [16].	Poor living conditions, (e.g., living with livestock), improper hygienic, and sanitary practices, (e.g., eating raw food or drinking raw, water) will increase the morbidity of STH [17,18].	Worldwide, more than 1.5 billion people (24% of the world’s population) are infected with STH, mainly including Ascariasis (819.0 million), *huriasTricis* (464.6 million), and hookworm infection (438.9 million). Nearly 70% of the global STH burden occur in Asia [4]. Results of China’s second national survey for STH showed that the prevalence of *A. lumbricoides* and *T. trichiura* were 12.7% and 4.6%, respectively [19].
*Trichuriasis*	*Adenophorea*	*Trichuris trichiura*	Immature eggs in soil under favorable conditions take about three weeks to mature. Humans are infected through contaminated water and soil [16].			
*Cryptosporidiosis*	*Apicomplexan*	*Cryptosporidium* spp.	*Cryptosporidium* is transmitted via contaminated water or food, swimming or bathing in surface waters [6].	Animal contact; Children from 1 to 9 years old; Hypoimmunity Both high ambient temperature and high rainfall. Malnutrition Overcrowding [20].	Inadequate sanitation and safe drinking water coverage will increase the transmission of *Cryptosporidium* [20].	*Cryptosporidiosis* is an important cause of diarrhea disease, more common in developing countries (5% to >10%) than in developed countries (<1–3%) [6]. In China, the proportion of diarrhea cases caused by *Cryptosporidium* varies between 1.4% to 10.4% and prevalence among the general population range from 0.79% to 6.59% [21].

STH: Soil-transmitted helminths.

**Table 2:** Distribution of demographic factors for participants included and not included in the analysis ^a^.

Characteristics	Participants Included in the Analysis (*n* = 321)	Participants not Included in the Analysis (*n* = 27)	*p*-Value
Age ^b^	12.47 ± 1.91	13.11 ± 1.85	0.093
Male sex	202 (62.93%)	12 (44.44%)	0.058
No. of family members (≥6)	254 (79.13)	19 (70.37%)	0.288

^a^ Data are number (%) of participants unless otherwise indicated, and using chi-square test. ^b^ Data are mean ± SD, and using 2-independent-samples *t*-test.

**Table 3:** Prevalence of *A. lumbricoides*, *T. trichiura*, *Cryptosporidium* and coinfections by age group ^a^.

Characteristics	Total	<12 Years	12–13 Years	>13 Years	*p*-Value
*A. lumbricoides* infection	10.0 (6.9–13.8)	12.4 (6.3–21.0)	13.0 (7.7–20.0)	4.0 (1.1–8.8)	0.039
*T. trichiura* infection	25.2 (20.6–30.4)	28.1 (19.1–38.6)	19.1 (12.7–26.9)	30.7 (21.9–40.7)	0.100
*Cryptosporidium* infection	2.4 (1.1–4.9)	2.2 (0.3–7.9)	3.8 (1.2–8.7)	1.0 (0.0–5.4)	0.394
Coinfection					
*A. lumbricoides*/*T. trichiura*	3.7 (1.9–6.4)	5.6 (1.8–12.6)	4.6 (1.7–9.7)	1.0 (0–5.4)	0.164
*A. lumbricoides*/*Cryptosporidium*	0.3 (0–1.7)	0	0.8 (0–4.2)	0	1.000
*T. Trichiura*/*Cryptosporidium*	0.9 (0.2–2.7)	0	1.5 (0.2–5.4)	1.0 (0–5.4)	0.784

^a^ Values are infection rates with 95% confidence intervals and compared using the Fisher’s exact test.

**Table 4:** Description of the participants by age group. Data are number (%) of participants unless otherwise indicated.

Characteristics	<12 Years (*n* = 89)	12–13 Years (*n* = 131)	>13 Years (*n* = 101)	Total (*n* = 321)
**Sex**				
Female	39 (43.82)	48 (36.64)	32 (31.68)	119 (37.07)
Male	50 (56.18)	83 (63.36)	69 (68.32)	202 (62.93)
**No. of family members**				
<6	18 (20.22)	24 (18.32)	25 (24.75)	67 (20.87)
≥6	71 (79.78)	107 (81.68)	76 (75.25)	254 (79.13)
**Ethnicity**				
Han	1 (1.12)	2 (1.53)	1 (0.99)	4 (1.25)
Yi	88 (98.88)	129 (98.47)	100 (99.01)	317 (98.75)
**Having domestic animals**				
No	1 (1.12)	0 (0.00)	5 (4.95)	6 (1.87)
Yes	88 (98.88)	131 (100.00)	96 (95.05)	315 (98.13)
**Drinking unboiled water at home**				
No or Occasionally	5 (5.62)	22 (16.79)	23 (22.77)	50 (15.58)
Always	84 (94.38)	109 (83.21)	78 (77.23)	271 (84.42)
**Water sources at home**				
Springs	70 (78.65)	117 (89.31)	88 (87.13)	275 (85.67)
Wells or rivers	19 (21.35)	14 (10.69)	13 (12.87)	46 (14.33)
**Washing hands before meals**				
No	11 (12.36)	7 (5.34)	5 (4.95)	23 (7.17)
Occasionally	62 (69.66)	90 (68.70)	81 (80.20)	233 (72.59)
Always	16 (17.98)	34 (25.95)	15 (14.85)	65 (20.25)
**Washing hands after defecation**				
No	36 (40.45)	27 (20.61)	13 (12.87)	76 (23.68)
Occasionally	43 (48.31)	64 (48.85)	62 (61.39)	169 (56.65)
Always	10 (11.24)	40 (30.53)	26 (25.74)	76 (23.68)
**Eating raw food at home**				
No	14 (15.73)	18 (13.74)	11 (10.89)	43 (13.40)
Yes	75 (84.27)	113 (86.26)	90 (89.11)	278 (86.60)
**Engaging in farm work**				
No	21 (23.60)	50 (38.17)	33 (32.67)	104 (32.40)
Yes	68 (76.40)	81 (61.83)	68 (67.33)	217 (67.60)
**Using human or animal feces as fertilizer**				
No	5 (5.62)	5 (3.82)	7 (6.93)	17 (5.30)
Yes	84 (94.38)	126 (96.18)	94 (93.07)	304 (94.70)
**Having a household lavatory at home**				
No	40 (44.94)	39 (29.77)	33 (32.67)	112 (34.89)
Yes	49 (55.06)	92 (70.23)	68 (67.33)	209 (65.11)
**Type of lavatory at home**				
No	40 (44.94)	39 (29.77)	33 (32.67)	112 (34.89)
Simple	34 (38.20)	61 (46.56)	42 (41.58)	137 (42.68)
Triple compartment or biogas pools	15 (16.85)	31 (23.66)	26 (25.74)	72 (22.43)
**Eating at the school canteen**				
No	2 (2.25)	5 (3.82)	4 (3.96)	11 (3.43)
Only lunch	10 (11.24)	7 (5.34)	7 (6.93)	24 (7.48)
Three meals	77 (86.52)	119 (90.84)	90 (89.11)	286 (89.10)
**Living on campus**				
No	12 (13.48)	12 (9.16)	11 (10.89)	35 (10.90)
Yes	77 (86.52)	119 (90.84)	90 (89.11)	286 (89.10)

**Table 5:** Univariable and multivariable analysis for the associations between potential risk factors and *A. lumbricoides* and *T. trichiura* infections.

Characteristics	No. of Tested (%)	*A. lumbricoides*					*T. trichiura*				
		No. of Infections (%)	cOR (95% CI)	*p*-Value	aOR (95% CI)	*p*-Value	No. of Infections (%)	cOR (95% CI)	*p*-Value	aOR (95% CI)	*p*-Value
**Sex**											
Female	119 (37.07)	16 (12.61)	1.00				31 (26.05)	1.00			
Male	202 (62.93)	17 (8.42)	0.64 (0.31–1.33)	0.236			50 (24.75)	0.93 (0.56–1.57)	0.794		
**Age group**											
<12	96 (29.91)	11 (12.36)	1.00				25 (28.09)	1.00			
12–13	140 (43.61)	17 (12.98)	1.06 (0.47–2.38)	0.902			25 (19.08)	0.60 (0.32–1.14)	0.124		
>13	117 (36.45)	4 (3.96)	0.29 (0.09–0.95)	0.037			31 (30.69)	1.13 (0.61–2.12)	0.699		
**No. of family members**											
<6	67 (20.87)	1 (5.97)	1.00				14 (20.90)	1.00			
≥6	254 (79.13)	28 (11.02)	1.95 (0.66–5.77)	0.224			67 (26.38)	1.36 (0.71–2.60)	0.365		
**Drinking unboiled water at home**											
No or Occasionally	50 (15.58)	4 (8.00)	1.00				9 (18.00)	1.00			
Always	271 (84.42)	28 (10.33)	1.32 (0.44–3.96)	0.649			72 (26.57)	1.65 (0.76–3.56)	0.202		
**Water sources at home**											
Springs	276 (85.98)	23 (8.33)	1.00		1.00		66 (23.91)	1.00			
Wells or rivers	45 (14.02)	9 (20.00)	2.67 (1.15–6.20)	0.033	2.67 (1.15–6.20)	0.033	15 (33.33)	1.59 (0.81–3.14)	0.224		
**Washing hands before meals**											
No	23 (7.17)	4 (17.39)	1.00				5 (21.74)	1.00			
Occasionally	233 (72.59)	19 (8.15)	0.42 (0.13–1.37)	0.179			60 (25.75)	1.25 (0.44–3.51)	0.703		
Always	65 (20.25)	9 (13.85)	0.76 (0.21–2.77)	0.677			16 (24.62)	1.18 (0.38–3.68)	0.806		
**Washing hands after defecation**											
No	76 (23.68)	10 (13.16)	1.00				17 (22.37)	1.00			
Occasionally	169 (56.65)	14 (8.28)	0.60 (0.25–1.41)	0.250			50 (29.59)	0.60 (0.25–1.41)	0.245		
Always	76 (23.68)	8 (10.53)	0.78 (0.29–2.09)	0.627			14 (18.42)	0.78 (0.29–2.10)	0.554		
**Eating raw food at home**											
No	43 (13.40)	6 (13.95)	1.00				10 (23.26)	1.00			
Yes	278 (86.60)	26 (9.35)	0.64 (0.25–1.65)	0.360			71 (25.54)	1.13 (0.53–2.41)	0.768		
**Engaging in farm work at home**											
No	104 (32.40)	7 (5.77)	1.00				28 (26.92)	1.00			
Yes	217 (67.60)	26 (11.98)	2.22 (0.89–5.58)	0.080			53 (24.42)	0.88 (0.52–1.49)	0.629		
**Using human or animal feces as fertilizer**											
No	17 (5.30)	1 (5.88)	1.00				2 (11.76)	1.00			
Yes	304 (94.70)	31 (10.20)	1.82 (0.23–14.17)	0.638			79 (25.99)	2.63 (0.59–11.77)	0.195		
**Having a lavatory at home**											
No	112 (34.89)	11 (9.82)	1.00				38 (33.93)	1.00		1.00	
Yes	209 (65.11)	21 (10.05)	1.03 (0.48–2.21)	0.962			43 (20.57)	0.50 (0.30–0.84)	0.010	0.50 (0.30–0.84)	0.010
**Types of lavatories at home**											
No	112 (34.89)	11 (9.82)	1.00				38 (33.93)	1.00			
Simple	137 (42.68)	13 (9.49)	0.96 (0.41–2.24)	0.927			28 (20.44)	0.50 (0.28–0.89)	0.018		
Triple compartment or biogas pools	72 (22.43)	8 (11.11)	1.15 (0.44–3.01)	0.777			15 (20.83)	0.51 (0.26–1.02)	0.057		
**Living on campus**											
No	35 (10.90)	3 (8.57)	1.00				7 (20.00)	1.00			
Yes	286 (89.10)	29 (10.14)	1.20 (0.35–4.18)	0.821			74 (25.87)	1.40 (0.59–3.33)	0.467		
**Eating at the school canteen**											
No	11 (3.43)	1 (9.09)	1.00				1 (9.09)	1.00			
Only lunch	24 (7.48)	2 (8.33)	0.91 (0.07–11.23)	0.918			6 (25.00)	3.33 (0.35–31.70)	0.323		
Three meals	286 (89.10)	29 (9.97)	1.13 (0.14–9.13)	0.996			74 (25.87)	3.49 (0.44–27.69)	0.225		
***A. lumbricoides***											
No	289 (90.03)	–	–				69 (23.88)	1.00			
Yes	32 (9.97)	–	–				12 (37.50)	1.91 (0.89–4.11)	0.142		
***T. trichiura***											
No	240 (74.77)	20 (8.33)	1.00				–	–			
Yes	81 (25.23)	12 (14.81)	1.91 (0.89–4.11)	0.107			–	–			
***Cryptosporidium***											
No	313 (97.51)	31 (9.90)	1.00				78 (24.92)	1.00			
Yes	8 (2.49)	1 (12.50)	1.30 (0.15–10.91)	0.757			3 (37.50)	1.81 (0.42–7.74)	0.441		

**Table 6:** Univariable and multivariable analysis for the association between potential risk factors and *Cryptosporidium* and any intestinal infection.

Characteristics	No. of Tested (%)	*Cryptosporidium*					Any intestinal Infection				
		No. of Infections (%)	cOR (95% CI)	*p*-Value	aOR (95% CI)	*p*-Value	No. of Infections (%)	cOR (95% CI)	*p*-Value	aOR (95% CI)	*p*-Value
**Sex**											
Female	119 (37.07)	4 (3.36)	1.00				43 (36.13)	1.00			
Male	202 (62.93)	4 (1.98)	0.58 (0.14–2.37)	0.448			62 (30.69)	0.78 (0.49–1.26)	0.316		
**Age group**											
<12	96 (29.91)	2 (2.25)	1.00				33 (37.08)	1.00			
12–13	140 (43.61)	5 (3.82)	1.73 (0.33–9.10)	0.520			38 (29.01)	0.69 (0.47–1.56)	0.210		
>13	117 (36.45)	1 (0.99)	0.44 (0.04–4.88)	0.500			34 (33.66)	0.86 (0.39–1.23)	0.623		
**No. of family members**											
<6	67 (20.87)	1 (1.49)	1.00				17 (25.37)	1.00			
≥6	254 (79.13)	7 (2.76)	1.87 (0.23–15.47)	0.561			88 (34.65)	1.56 (0.85–2.87)	0.152		
**Water sources at home**											
Wells or rivers	45 (14.02)	1 (2.22)	1.00				20 (44.44)	1.00			
Springs	276 (85.98)	7 (2.54)	1.18 (0.14–9.78)	0.881			85 (30.80)	0.52 (0.28–0.99)	0.046	0.52 (0.28–0.99)	0.046
**Drinking unboiled water at home**											
No or Occasionally	50 (15.58)	2 (4.00)	1.00				13 (26.00)	1.00			
Always	271 (84.42)	6 (2.14)	1.84 (0.36–9.39)	0.463			92 (33.95)	1.46 (0.74–2.89)	0.273		
**Wash hands before meals**											
No	23 (7.17)	1 (4.35)	1.00				9 (39.13)	1.00			
Occasionally	233 (72.59)	6 (2.58)	0.58 (0.07–5.05)	0.623			75 (32.19)	0.74 (0.31–1.78)	0.500		
Always	65 (20.25)	1 (1.54)	0.34 (0.02–5.73)	0.457			21 (32.31)	0.74 (0.28–1.99)	0.554		
**Wash hands after defecation**											
No	76 (23.68)	2 (2.63)	1.00				26 (34.21)	1.00			
Occasionally	169 (56.65)	2 (1.18)	0.44 (0.06–3.21)	0.420			57 (33.73)	0.98 (0.55–1.73)	0.941		
Always	76 (23.68)	4 (5.26)	2.06 (0.37–11.57)	0.414			22 (28.95)	0.79 (0.45–1.40)	0.486		
**Eating raw food at home**											
No	43 (13.40)	2 (4.65)	1.00				16 (37.21)	1.00			
Yes	278 (86.60)	6 (2.16)	0.45 (0.09–2.32)	0.341			89 (32.01)	0.76 (0.41–1.55)	0.500		
**Engaging in farm work**											
No	104 (32.40)	2 (1.92)	1.00				32 (30.77)	1.00			
Yes	217 (67.60)	6 (2.76)	1.45 (0.29–7.31)	0.652			73 (33.64)	1.14 (0.69–1.89)	0.877		
**Using human or animal feces as fertilizer**											
No	17 (5.30)	0 (0.00)	1.00				3 (17.65)	1.00			
Yes	304 (94.70)	8 (2.63)	1.00 (0.06–18.10)	0.999			102 (33.55)	2.36 (0.66–8.39)	0.186		
**Having a lavatory at home**											
No	112 (34.89)	3 (2.68)	1.00				46 (41.07)	1.00			
Yes	209 (65.11)	5 (2.39)	0.89 (0.21–3.80)	0.875			59 (28.23)	0.56 (0.35–0.91)	0.020		
**Types of lavatories at home**											
No	112 (34.89)	3 (2.68)	1.00				46 (41.07)	1.00			
Simple	137 (42.68)	3 (2.19)	0.81 (0.16–4.11)	0.803			38 (27.74)	0.55 (0.32–0.94)	0.028		
Triple compartment or biogas pools	72 (22.43)	2 (2.78)	1.04 (0.17–6.37)	0.968			21 (29.17)	0.59 (0.31–1.11)	0.103		
**Living on campus**											
No	35 (10.90)	4 (11.43)	1.00				12 (34.29)	1.00			
Yes	286 (89.1)	4 (1.40)	0.11 (0.03–0.46)	0.003			93 (32.52)	0.92 (0.44–1.94)	0.833		
**Eating at the school canteen**											
No	11 (3.43)	2 (18.18)	1.00		1.00		3 (27.27)	1.00			
Only lunch	24 (7.48)	2 (8.33)	0.41 (0.05–3.37)	0.409	0.41 (0.05–3.37)	0.409	9 (37.50)	1.60 (0.34–7.64)	0.556		
Three meals	286 (89.10)	4 (1.25)	0.06 (0.01–0.40)	0.003	0.06 (0.01–0.40)	0.003	93 (32.52)	1.28 (0.33–4.96)	0.716		
***A. lumbricoides***											
No	289 (90.03)	7 (2.42)	1.00				–	–			
Yes	32 (9.97)	1 (3.13)	1.30 (0.16–10.91)	0.809			–	–			
***T. trichiura***											
No	240 (74.77)	5 (2.08)	1.00				–	–			
Yes	81 (25.23)	3 (3.7)	1.81 (0.42–7.74)	0.425			–	–			
